# A PDMS/MWCNTs RFID flexible tag with advanced resonator design for read range enhancement in IoT monitoring systems

**DOI:** 10.1038/s41598-025-86773-7

**Published:** 2025-03-20

**Authors:** Hafsa Anam, Syed Muzahir Abbas, Iain B. Collings, Subhas Mukhopadhyay

**Affiliations:** School of Engineering, Faculty of Science and Engineering, Macquarie University, Sydney, 2109 Australia

**Keywords:** Engineering, Nanoscience and technology

## Abstract

Low-cost and passive identification systems are necessary for real time IoT (internet of things) monitoring. The paper presents a novel designed chipless RFID tag towards read range enhancement of (2.70 mm). A novel approach of array-based resonators towards real time read range enhancement is proposed in this research work with efficient performance. A mould-based solution casting technique is used for chipless tag fabrication. Multi-Walled Carbon Nanotubes (MWCNTs) are used as electrodes with flexible Polydimethylsiloxane (PDMS) substrate. The developed battery-free tag can encode 3-bits data while staying in compact dimensions of 20 × 20mm^2^. The single unit tag is extended to 3 × 3-array structure. The novel approach leads the tag towards RCS increment of 25.17dBsm. The tag has been extended to 5 × 5-array for Poly-ethylene terephthalate (PET) substrate with silver nanoparticle-based ink radiator leading to RCS increment of 25.16dBsm. The tags read range performance is very highly reliable and stable as measured from multiple samples and is suitable for deployment in a smart sensing and IoT identification network.

## Introduction

Flexible passive chipless radio frequency identification (RFID) is one of leading technologies for internet of things (IoT) monitoring systems. Unlike traditional RFID systems that depend on microchips for data storage and transmission, chipless RFID employs alternative techniques such as electromagnetic resonators or frequency-modulated backscattering to transmit information. This method eliminates the need for battery-powered chips, resulting in more cost-effective solutions^1,2^. Integrating chipless RFID into IoT systems presents a promising opportunity to enhance connectivity and data exchange across various applications, including supply chain management and smart inventory systems. By deploying the advantages of chipless RFID, IoT solutions can achieve improved scalability, lower costs, and greater efficiency in tracking and identifying objects within a connected network^3–5^.

Passive Chipless RFID tags have two classifications, (1) time domain-based tags (TD) and (2) frequency domain-based tags (FD). The FD-based tags have preference over the TD-based tags, because the TD-based tags extremely complex structural designs with very large sizes. In FD-based tags, the encoded data bits are represented by the presence or absence of a specific resonant frequency^6^. Both the tag domains trade-off among coding capacity and read range. TD tags offer a greater read range but have a lower coding capacity compared to FD tags, which, on the other hand, provide a larger coding capacity but have a smaller read range^7^. FD-based tags are further classified into, (1) backscattered based tags, (2) retransmission-based tags. In backscattered-based tags also referred as radar cross section-based tags (RCS), the design/geometry of the tag resonator determines whether an incoming/incident electromagnetic (EM) wave is reflected or absorbed. The radar cross section (RCS) measures the EM response of the tags. Data is read as presence or absence of a resonance. A resonant peak indicates a logic 1, while its absence represents a logic 0. The retransmission-based tags have larger design, which comprises of resonant geometric structures and antennas^6^.

RFID systems are commonly used for identification and tracking across various industries. However, integrating sensor data with RFID data enhances the capabilities and applications of these technologies. In passive RFID systems, a reader wirelessly delivers energy to a tag as electromagnetic (EM) wave. The tag utilizes this energy to demodulate the incoming data and power any connected components. Then the tag transmits information such as its Identification (ID) or sensor data by modulating the signal through backscattering. Then the retransmitted signal is read by reader and received at system^8^. Passive RFID systems don’t need a battery, making these sensors cost-effective and providing them with extended autonomy. Despite these advantages, the primary drawback of a fully passive system is its requirement to be near the reader to receive the necessary power for the tag. This limitation restricts the communication range of the tag and reduces the number of possible applications^8^.

The key challenges in the chipless technology are simultaneously maintenance of major factors data capacity, tags size, and the read range. The required factors are also high spatial density and larger spectral density^9^. Apart from these, data encoding and decoding, manufacturing complexity, environmental sensitivity, signal interference, integration with existing system, durability and longevity are several considered challenges in the chip-free identification and sensing technology. In past, sufficient research has been done on many of these factors including data capacity, tags orientation, dimensions/size of tag. But one of the major challenges, an important aspect of chipless RFID tags, namely achieving a large read range, has not yet been thoroughly investigated. In^10^, the author has presented an interesting 20-bit chipless array approach towards improved magnitude and robustness, but the array has not been thoroughly investigated towards impact on read range. In^11^, BER Performance is evaluated, and array structure is analysed but it is not evaluated towards read range performance outcomes^12^. The authors of^13^have also utilized array concept for enhanced coding capacity with decreased mutual coupling, however the reads range or RCS enhancement is not considered. Various researchers are working on smart machine learning approaches towards better encoding^14–16^. Similarly, novel approach towards robust identification in chipless RFID tags via utilization of deep-learning (DP) and machine learning (ML) is an interesting work^17^. Extensive work is done by researchers for data capacity enhancements in chipless tags^18,19^. Also, sensor based chipless RFID tags are under extensive improvement through varying techniques^5,20–26^. However, these studies have not addressed the issue of read range in chipless systems, which is a problem that needs to be properly resolved for effective smart RFID tags.

The proposed work presents a solution towards simultaneous achievement of multi-factors, reasonable data capacity, compact size, flexible tags, and most importantly an improved read range. The work presents an approach towards the read range enhancement, which is a critical challenge due to the passive nature of these tags. We have created new resonator design that extend the read range by boosting signal strength and clarity. This involves optimizing the geometry and materials of the resonators to enhance their efficiency and resonance properties. Incorporating multiple resonators into a single tag can generate more distinct and powerful signals, which improves the read range. We have investigated methods for integrating and aligning multiple resonators to optimize the tag’s performance. A key technique for enhancing read range is the use of array arrangements. For a chipless RFID tag to achieve long-range readability, it needs to exhibit a higher radar cross section (RCS) response. Array-based chipless RFID tag presents a cost-effective solution towards read range enhancement, but also exhibits a trade-off between tags size and read range performance. The proposed chipless RFID tag features an extended read range and a high RCS response. The proposed single-unit tag has 3-bit data with an RCS increment from (−55.72dBsm to −30.55dBsm) in frequency range of (1.35 –6.9 GHz). Also, through this study, we have proposed a new approach to fabricate flexible sensor tags. The rest of the paper is arranged as follows: The designed tag and approach are illustrated in Sect. 2. Section 3 presents the fabrication of proposed chipless tags. The experimental results and performance evaluation are presented in Sect. 4. The RCS results are analysed in Sect. 5 whereas, read range calculation and discussion are made in Sect. 6 respectively. The conclusion is drawn in the last Sect. 7.

## Materials and process

**Fig. 1:**
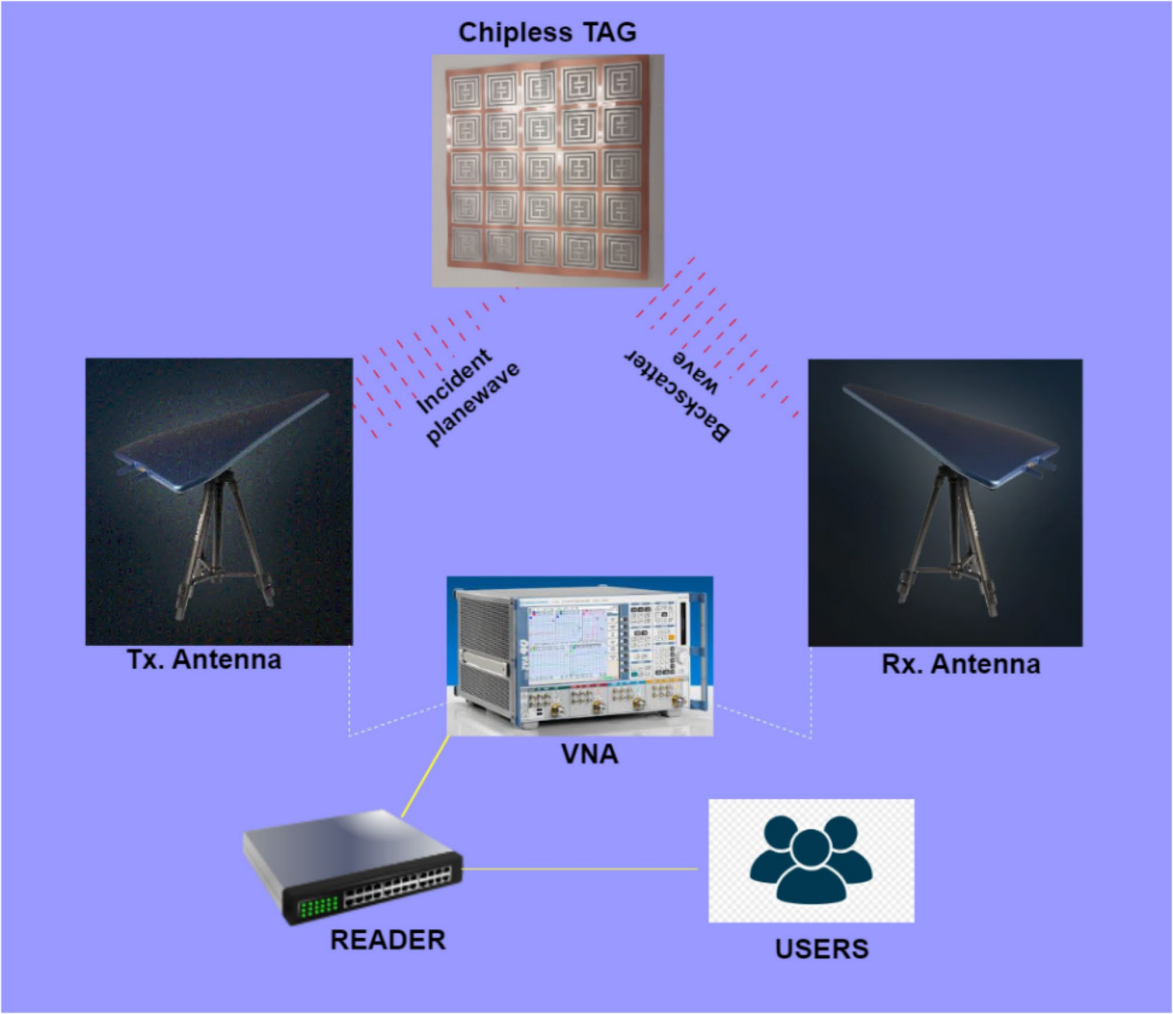
A typical CRFID system.

Proposed chipless RFID tag is newly designed resonator-based structure. The tag operates via backscattering of incident plane wave. Chipless RFID systems are a specific type of passive RFID system where the tag does not need both a power source and electronic components. Instead, the tag’s information is retrieved through its electromagnetic (EM) scattering response from data encoded in its structure^27,28^. Figure 1 illustrates working principle of chipless RFID tags with reader setup.

### Design of proposed CRFID tag

**Fig. 2:**
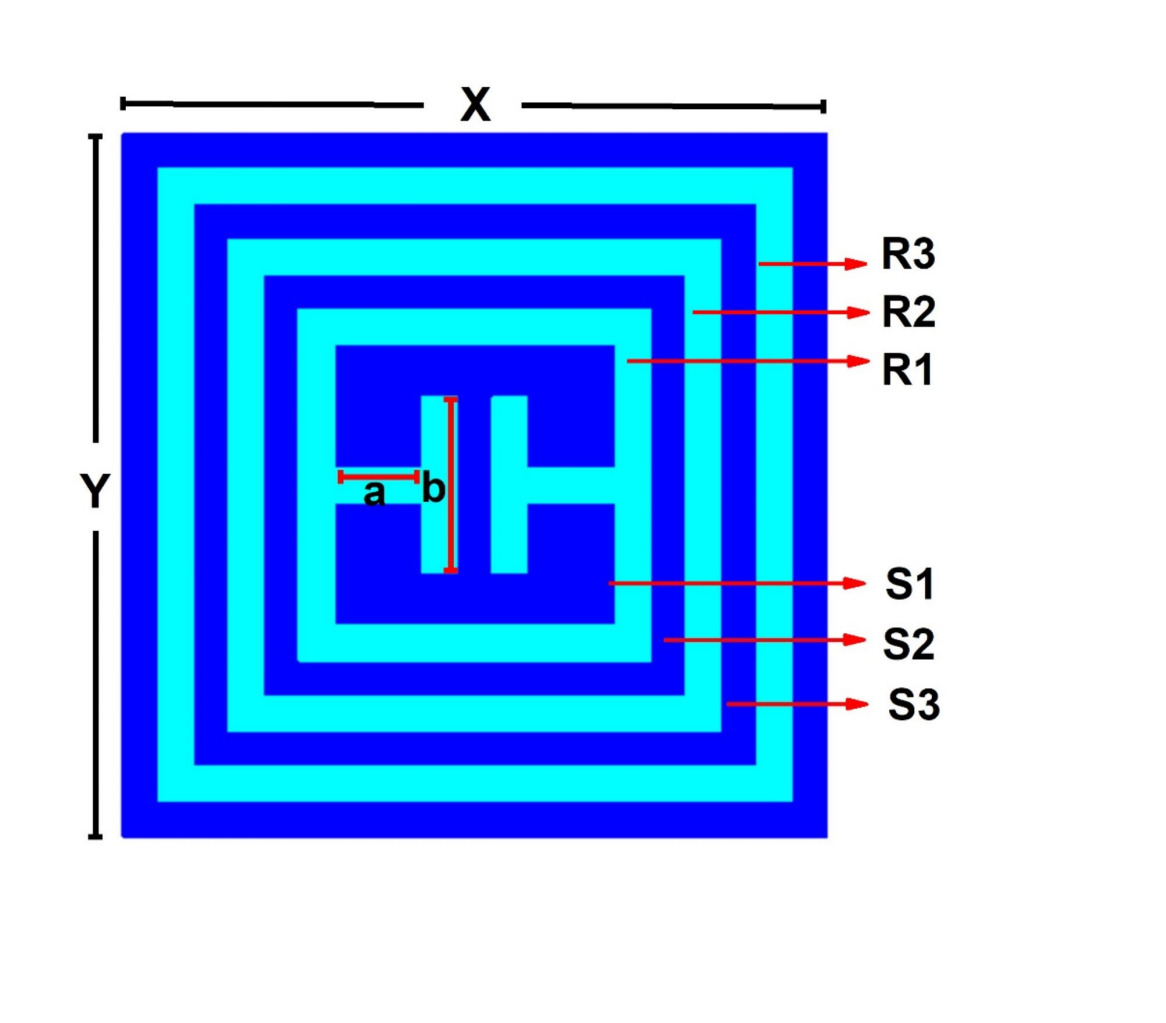
Proposed Chipless RFID Tag.

In this work, a new approach is developed and proposed for read range enhancement. The tag has been designed using PDMS substrate along with MWCNTs as electrode. Figure 2shows the layout of proposed 3-bit chipless RFID tag. The tag designed using substrate Polydimethylsiloxane (PDMS) along with MWCNTs as electrode/radiator is referred to as ‘Tag-A’. Whereas tag designed with PET and silver nanoparticles is ‘Tag-B’. PDMS is a flexible material, very suitable for flexible sensor designing. PDMS Substrate has 1 mm thickness, electrical conductivity = 0.03(S/m), Ɛ=2.88, and loss tangent = 0.03^29^. The radiator is composed of MWCNTs having 1 mm thickness, conductivity = 1 × 10^4^(S/m) respectively. Whereas PET has 0.1 mm thickness, Ɛ=2.9, tang = 0.0025 and silver has 0.015 mm thickness, conductivity = 9e + 06(S/m)^30^. Table 1 illustrates the dimensions of designed RFID tag with radius of slots and resonators labelled as S1, S2, S3, R1, R2, R3 respectively.

**Table 1:** Parameters of proposed single-unit RFID Tag.

Parameter	X	Y	a	b	R1	R2	R3	S1	S2	S3
Value (mm)	20	20	2.5	5	5	7	9	4	6	8

### Design of proposed approach array-based CRFID tag

A chipless RFID tag is essentially a metal component without any embedded intelligence for communication with the reader^31^. Therefore, chipless RFID needs extensive research and approaches towards better performance with enhanced read range. The read range of chipless RFID tags is one of the major challenges the technology faces in real-time deployment in IoT networks. Chipless RFID applications remain under discussion because of their reduced reading reliability under both static and dynamic conditions^31^. To address this issue, instead of using high-cost solutions, we have proposed a new approach to improve the read range without any high-cost electronic components.

**Fig. 3:**
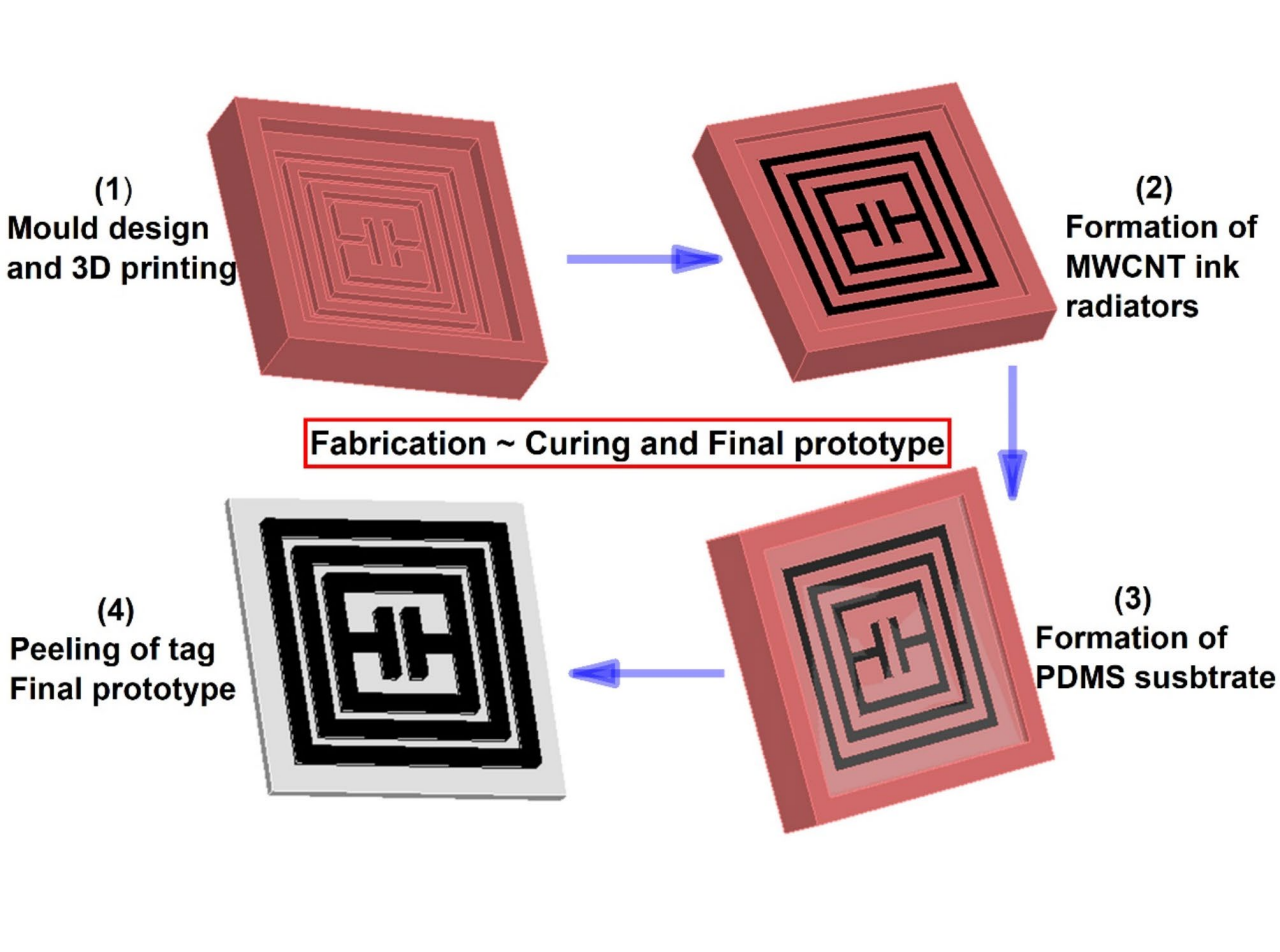
Fabrication Schematic of Tag-A.

The proposed tag is further been optimized towards 2 × 2-array design and its results are analysed. Then further optimization and analysis take us to next step of 3 × 3-array, where results are further enhanced. Tag-A has been visualized for 1-unit, 2 × 2-array and 3 × 3-array structures. Tag-B has been optimized further till 4 × 4-array and 5 × 5-array structures. Table 2 illustrates the dimension parameters of both Tag-A and Tag-B for single and array structures. Figure 3 shows the layout of proposed Tag-A fabrication schematic.

**Table 2:** Dimension parameters of proposed RFID tags.

Parameter	Tag-A 1-Unit	Tag-A 2 × 2	Tag-A 3 × 3	Tag-B 1-uniT	Tag-B 2 × 2	Tag-B 3 × 3	Tag-B 4 × 4	Tag-B 5 × 5
X (mm)	20	40	60	20	40	60	80	100
Y (mm)	20	40	60	20	40	60	80	100

## Materials and methods

This section presents the fabrication of proposed designed chipless RFID tags. The proposed tags have been fabricated through mould-based solution casting technique. After the fabrication, the tags are tested, and results are measured via vector network analyser and transmitter/receiver antennas. The samples are tested multiple times with 5 samples measurements per fabricated tag. Following is stepwise process from design to measurement of results.

- 1-unit Tag design.
- Array-based Tag design.
- Mould fabrication process.
- Chipless RFID Tag fabrication process.
- Experiments and measurement of response of proposed Tags.

**Fig. 4:**
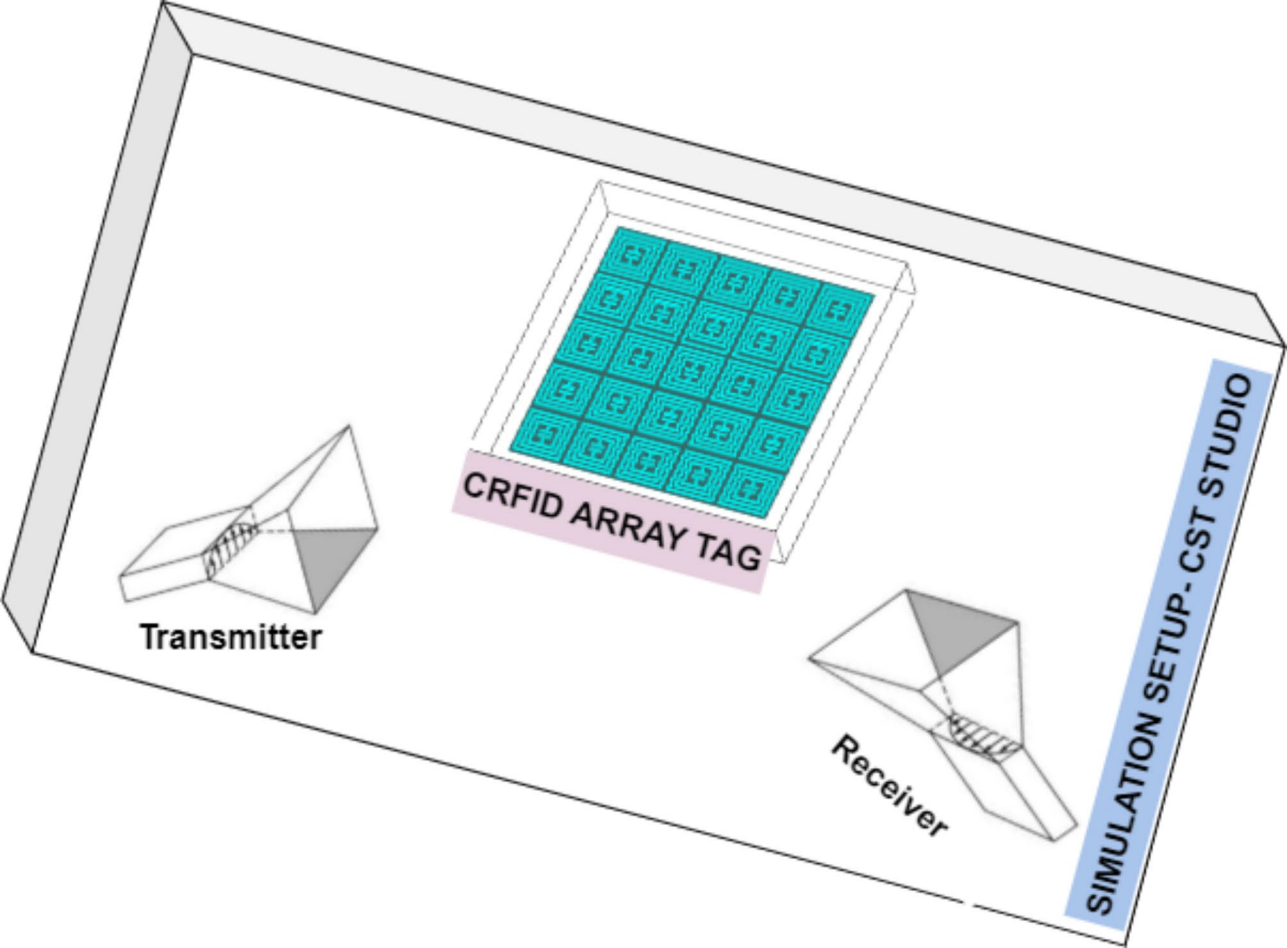
Simulation Set-up proposed Chipless tag.

### Design and fabrication of mould

CST Studio suit has been used for proposed tags design. The designed tags are further worked on towards mould design and fabrication using 3D-printing technique. The mould is initially designed using CST Studio suit software and further verified via design on Autodesk Fusion 360 software too. The mould is fabricated using Poly Lactic Acid (PLA) filament with 50% infill in Dreamer 3D-printer. Figure 4 shows simulation setup of proposed designed RFID tag. In CST Studio suit, after the tag design, frequency range is defined. Then, the probes are set at far-field distance from surface of tag. Far-field distance is calculated using largest dimensions of tag. The probes send and receive signal from tag and capture the electromagnetic signal as plot of RCS vs. frequency as output.

**Fig. 5:**
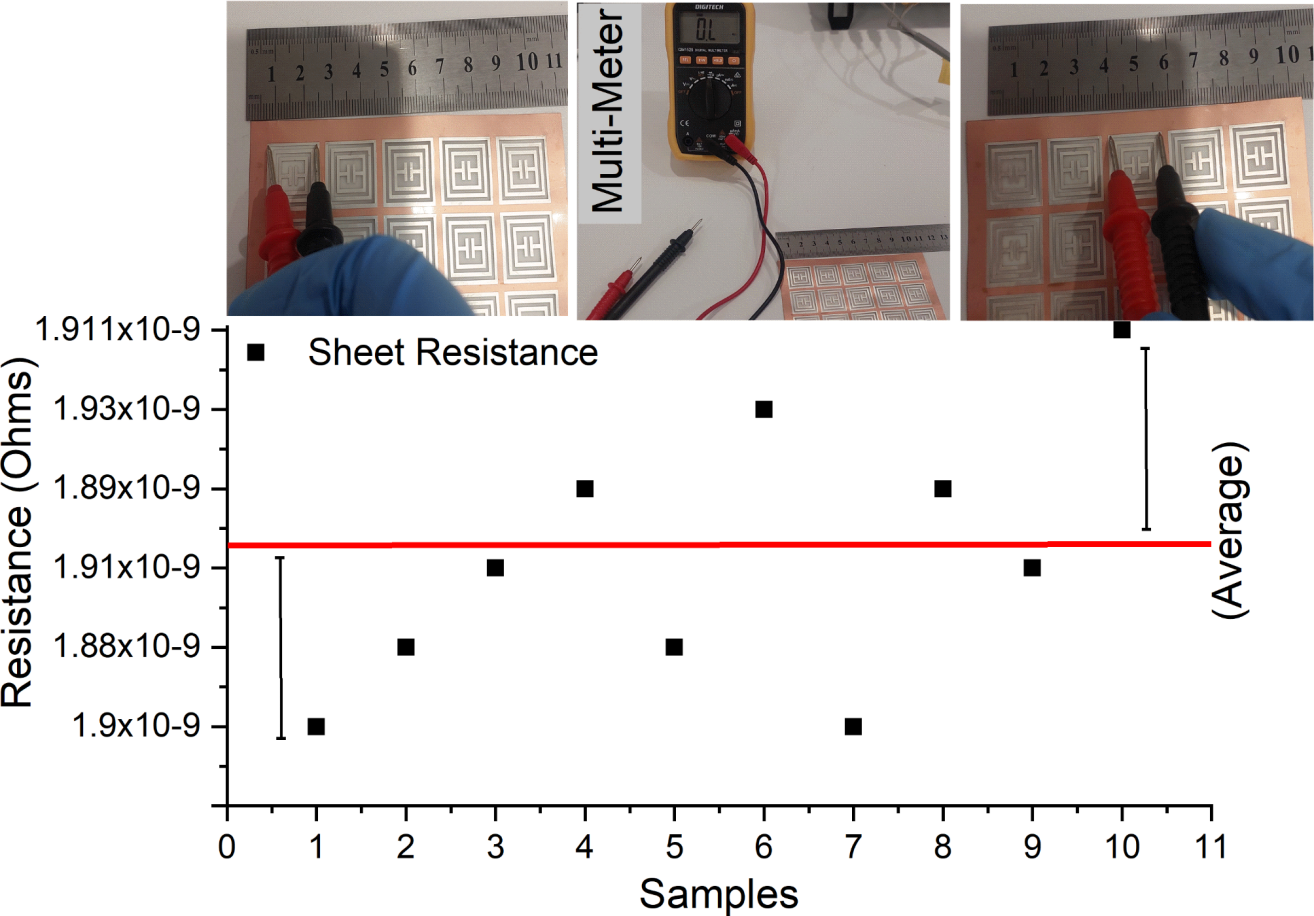
Sheet Resistance of Fabricated samples.

### Fabrication of single unit chipless transponder

**Fig. 6:**
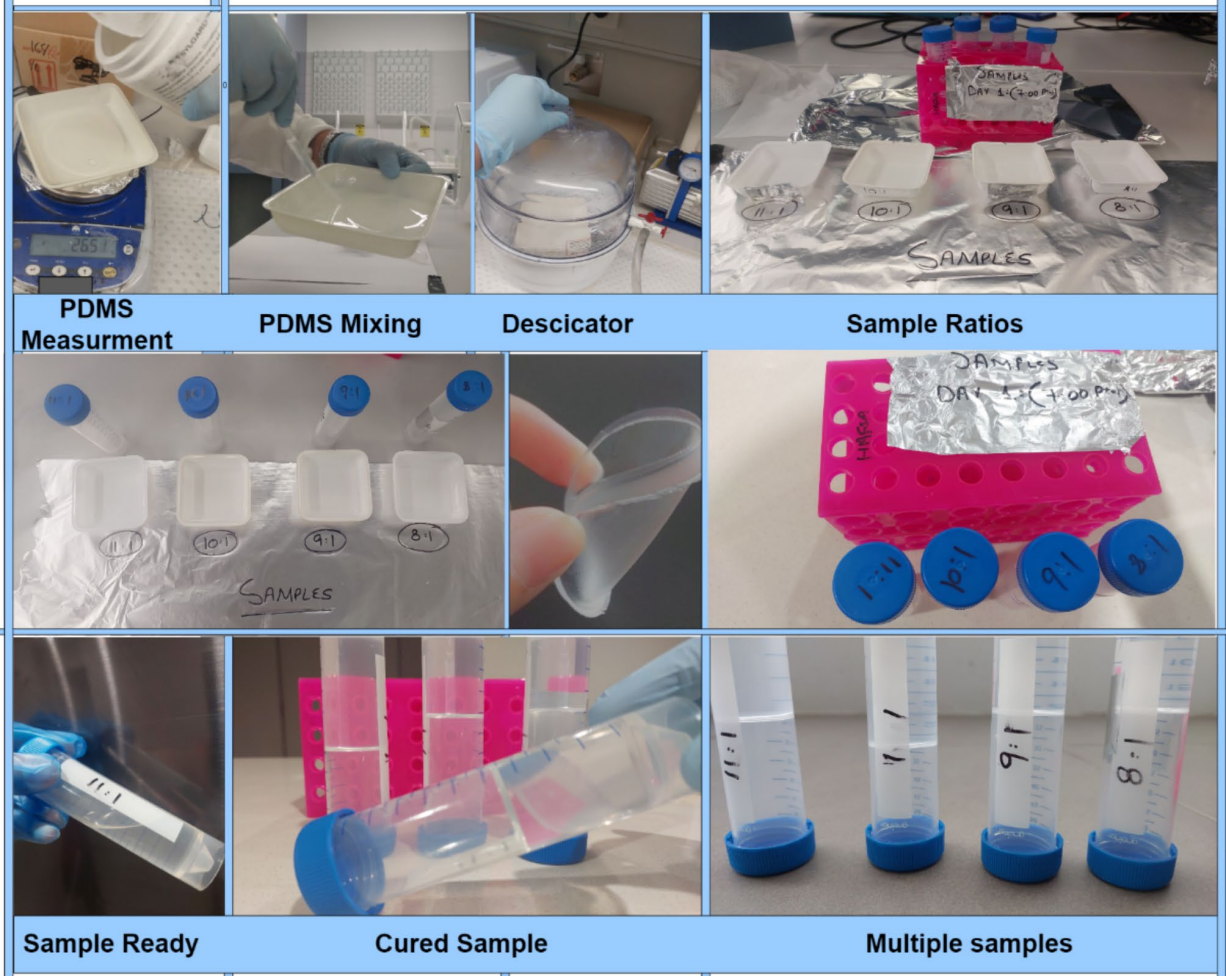
PDMS Ratio Testing for different samples.

The designed single-unit tag is optimized and fabricated in lab via mould casting technique. The fabrication process is simple and involves multiple steps of substrate and electrode preparation and curing processes. For fabrication, the silicone elastomer base and curing agent are mixed in 10:1 ratio for substrate solution. Initially, to check best flexibility of PDMS solution, it is prepared for varying ratios of 8:1, 9:1, 10:1 and 11:1. Than at the end, 10:1 is opted as best flexibility for desired PDMS substrate^32,33^. The sheet resistance of fabricated samples is tested using multi-meter. We have used two-point probe method using multi-meter to check resistance of sample at various points. We have kept the distance of 0.5 mm constant between the probes and tested the value at various points with 10 samples. Then, an average is acquired, and graph plot is shown in Fig. 5. Figure 6 shows PDMS Ratio Testing for different samples process.

**Fig. 7:**
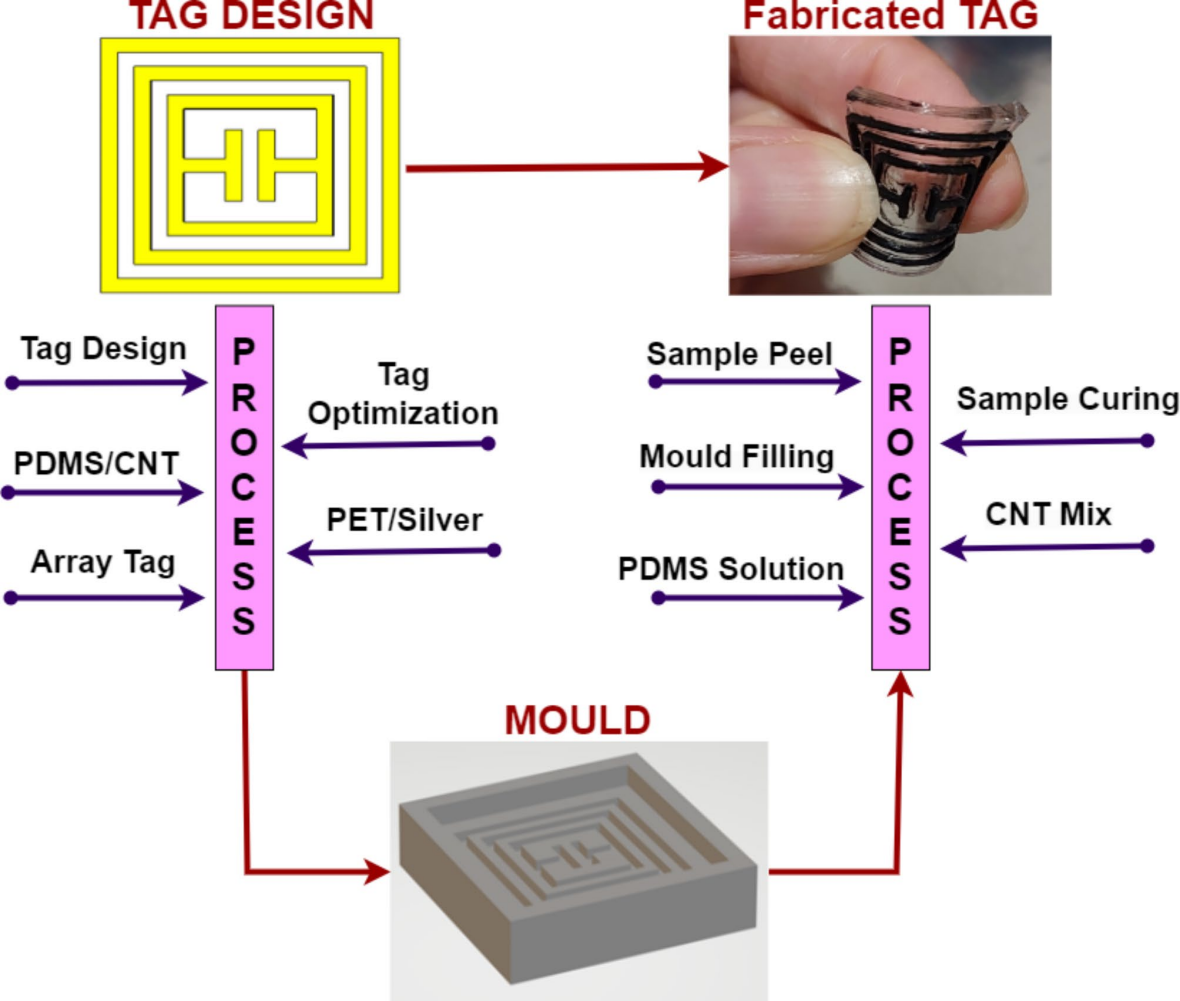
Schematic of RFID tag Fabrication.

**Fig. 8:**
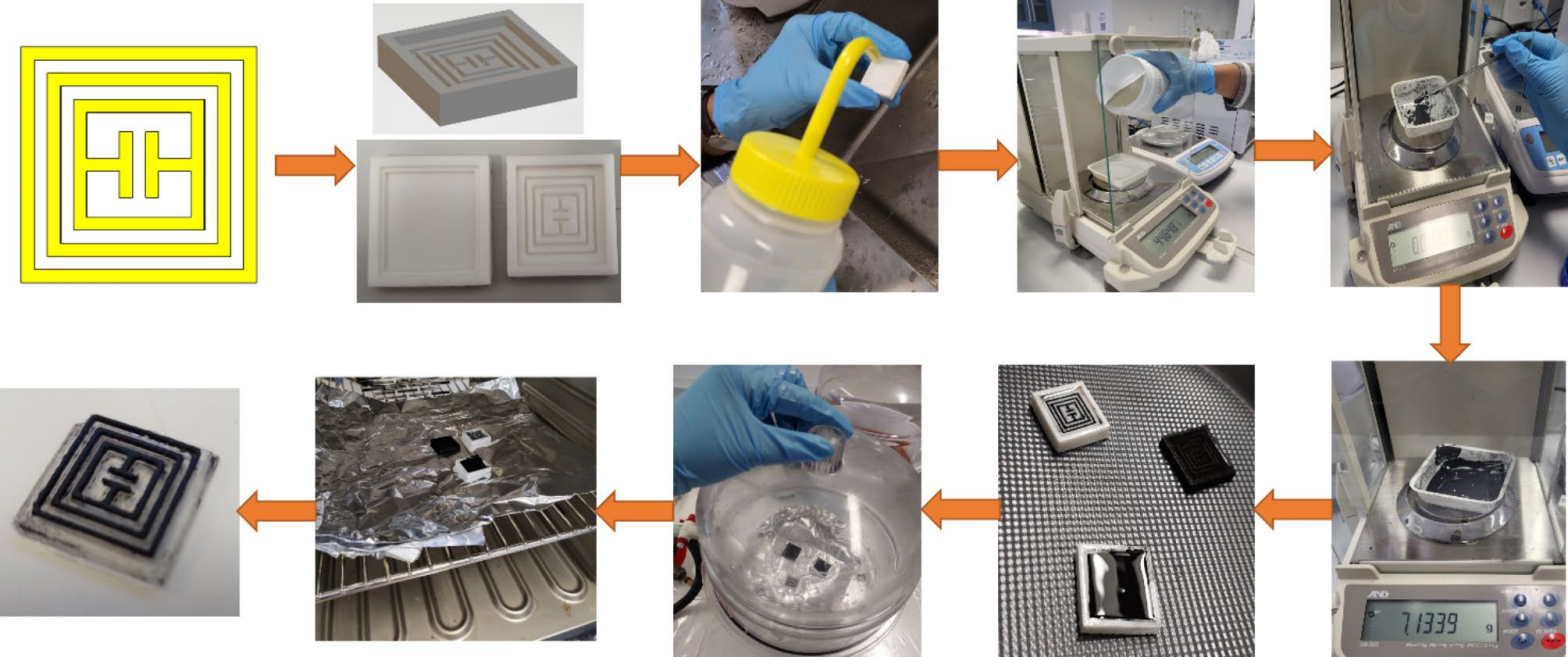
Fabrication of Single unit Proposed RFID tag.

Then the PDMS solution is passed through desiccator for removing any trapped air bubbles. After that, the conductive ink for electrode is prepared. PDMS (10:1) solution is mixed in MWCNTs powder. Ultrasonication and high-speed mechanical stirring are used for ink preparation. To reduce MWCNTs agglomeration, an ambient environment is opted for ultrasonication. Then, the process is followed by mechanical stirring for 30 min at 500 rpm speed. Next step is conductive ink casting onto the mould. Then, the moulds are cured for 3 h at 60 °C. At the end, PDMS solution is pored over the mould. Care should be taken for balancing the height of substrate, as it can be uneven. To balance the top layer, casting knife is used carefully for even surface. Then the samples are first placed in the desiccator to remove any trapped air bubbles inside the solution. At the last, samples are cured for 5 h at 60 °C. Once the samples are cured and solidified, the prepared tags are peeled off and ready for experiments. The step-by-step prototyping schematic is shown in Fig. 7. Figure 8 illustrates the single unit chipless RFID tag fabrication process step by step.

### Fabrication of array-based Chipless transponder

**Fig. 9:**
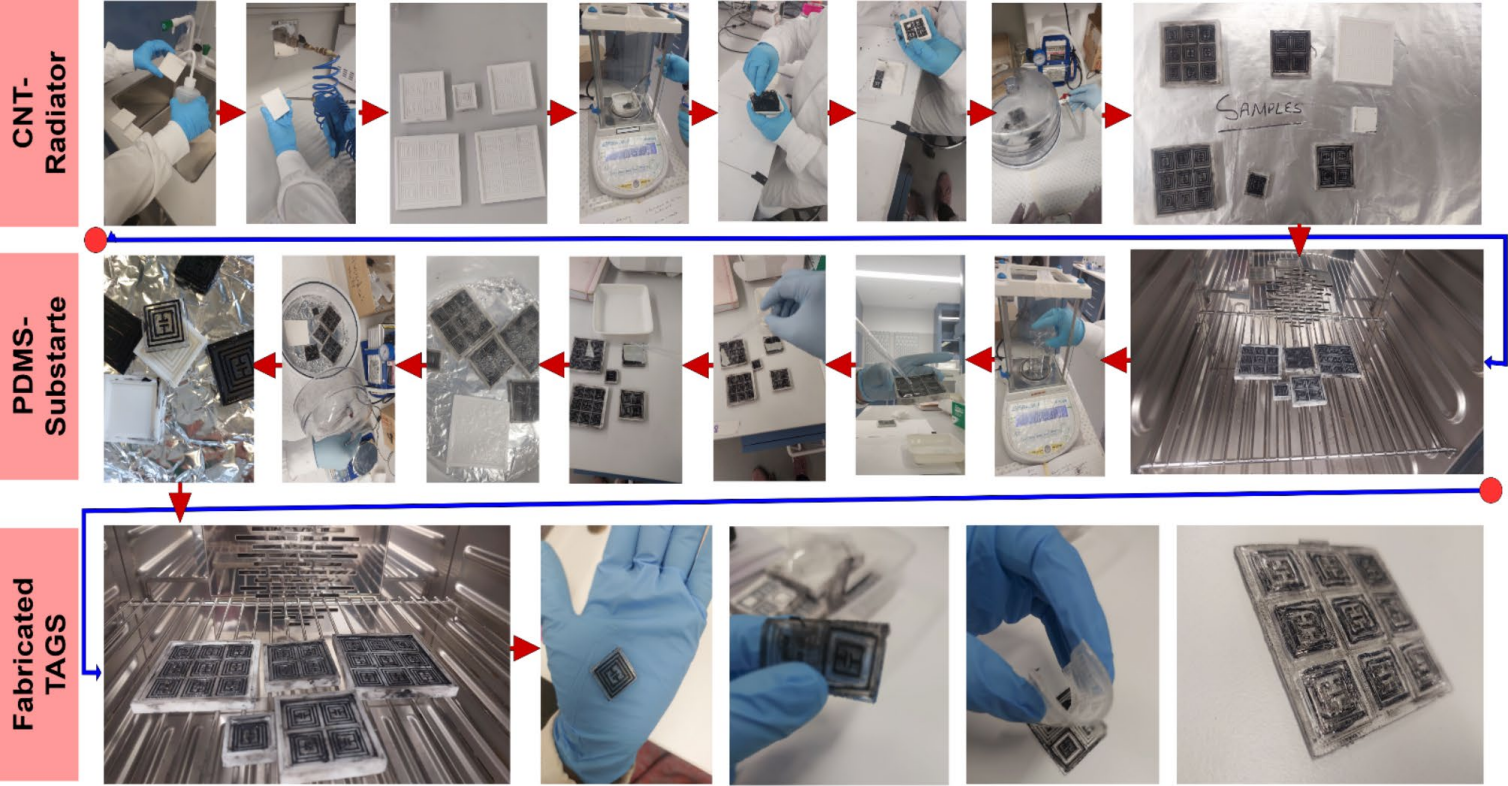
Fabrication of Proposed Array-based RFID tag.

The novel approach towards real time read range enhancement is based on design of array-based chipless RFID tag. The proposed PDMS/MWCNTs based tag has been designed and fabricated for 2 × 2-array and 3 × 3-array structures respectively. Figure 9 shows in detail the fabrication process of array-based chipless tags. The same process is followed as described in previous section for single-unit tag fabrication. Herein also, PDMS solution is prepared at 10:1 for silicone elastomer base and curing agent. Then, the array-based moulds are followed through conductive ink casting process and cured at 60 °C for 3 h. Then, PDMS susbtarte is poured at the top of cured electrode and trapped air bubbles vanished via desiccator followed by curing at 60 C again for 5 h. At the end, prepared 2 × 2-array and 3 × 3-array tags are peeled off and further examined and read by experimental process ahead.

## Experimental process

**Fig. 10:**
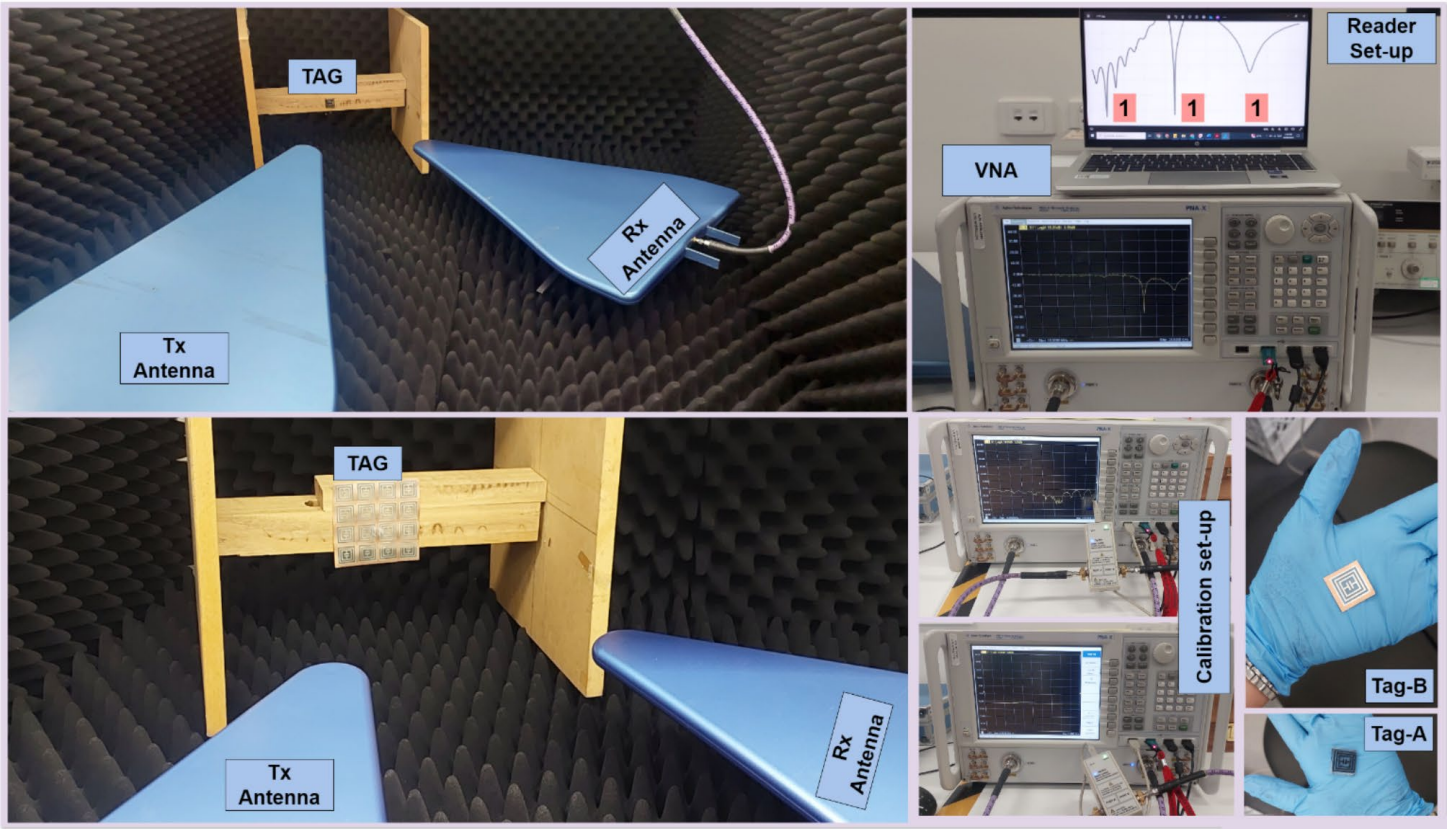
Experimental Setup for CRFID Tag detection/reading (PENDING).

Proposed work is tested, and results are measured using VNA along with transmitter and receiver antennas for data encoding and decoding. Figure 10 show the experimental setup for testing proposed tags. The tag is kept at far-field distance from two antennas and readings are measured as radar cross section RCS (dBsm) vs. frequency (GHz). Every fabricated is tested and results are measured 5 times/sample for accuracy and reliability.

The VNA analyser that we have used for experiments and measurement is Agilent Technologies PNA-X (N5242A) Analyzer operational at 10 MHz to 26.5 GHz frequency range. For RS magnitude measurements, we have used log periodical antennas as transmitter and receiver antennas. HyperLOG^®^ 4060 are the used log periodical antennas with 6dBi gain, 50Ω nominal impedance, VSWR < 2 and (590 × 360 × 30 mm) dimensions. In the experiment, initially 50Ω SMA cables are connected through VNA with calibration device. The electronic calibration module is Agilent (N-4691–60006) operational at 300 kHz-26.5 GHz, with (± 10 V DC/ +10 dBm MAX).

**Fig. 11:**
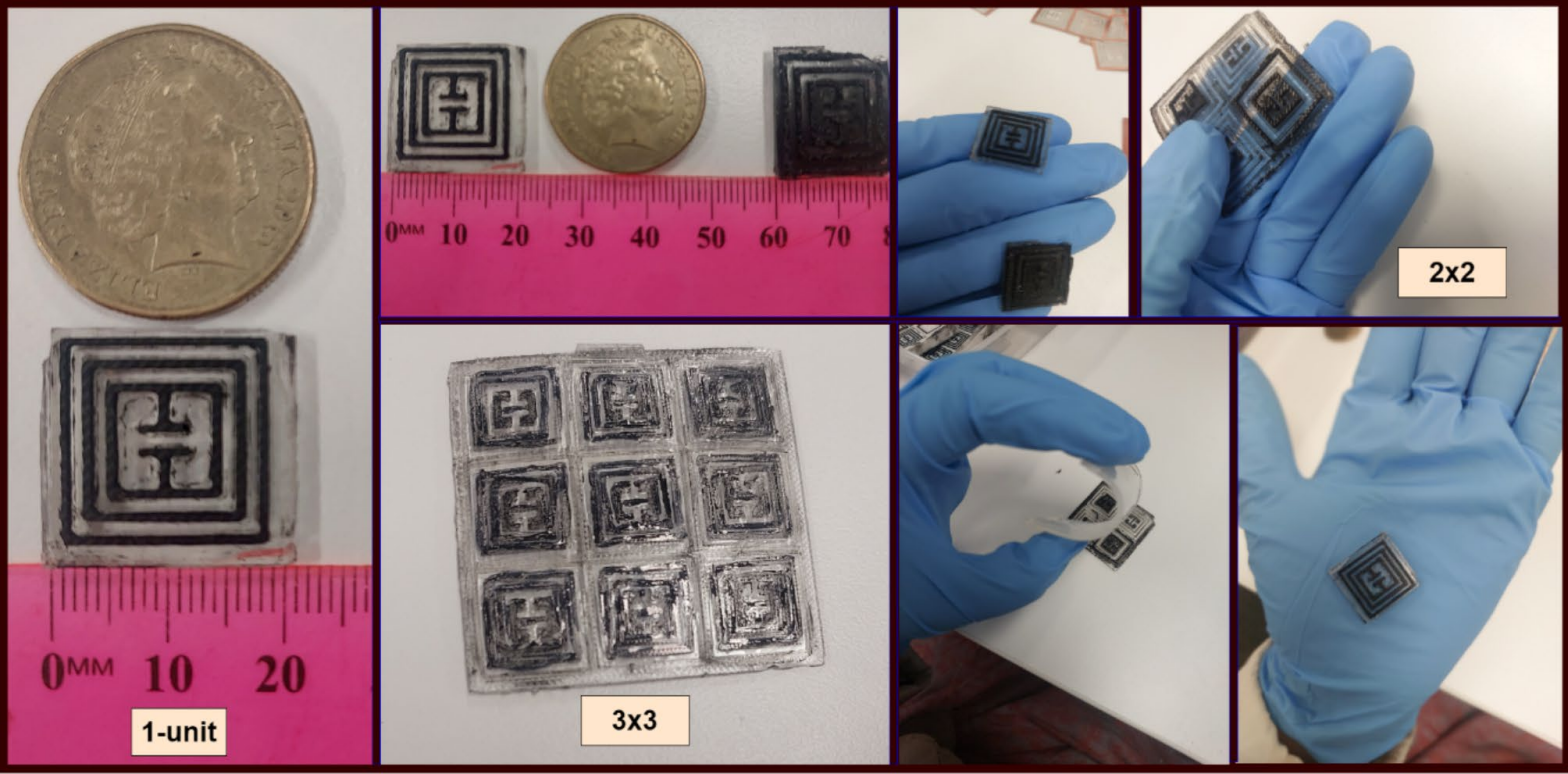
Fabricated samples Tag-A.

**Fig. 12:**
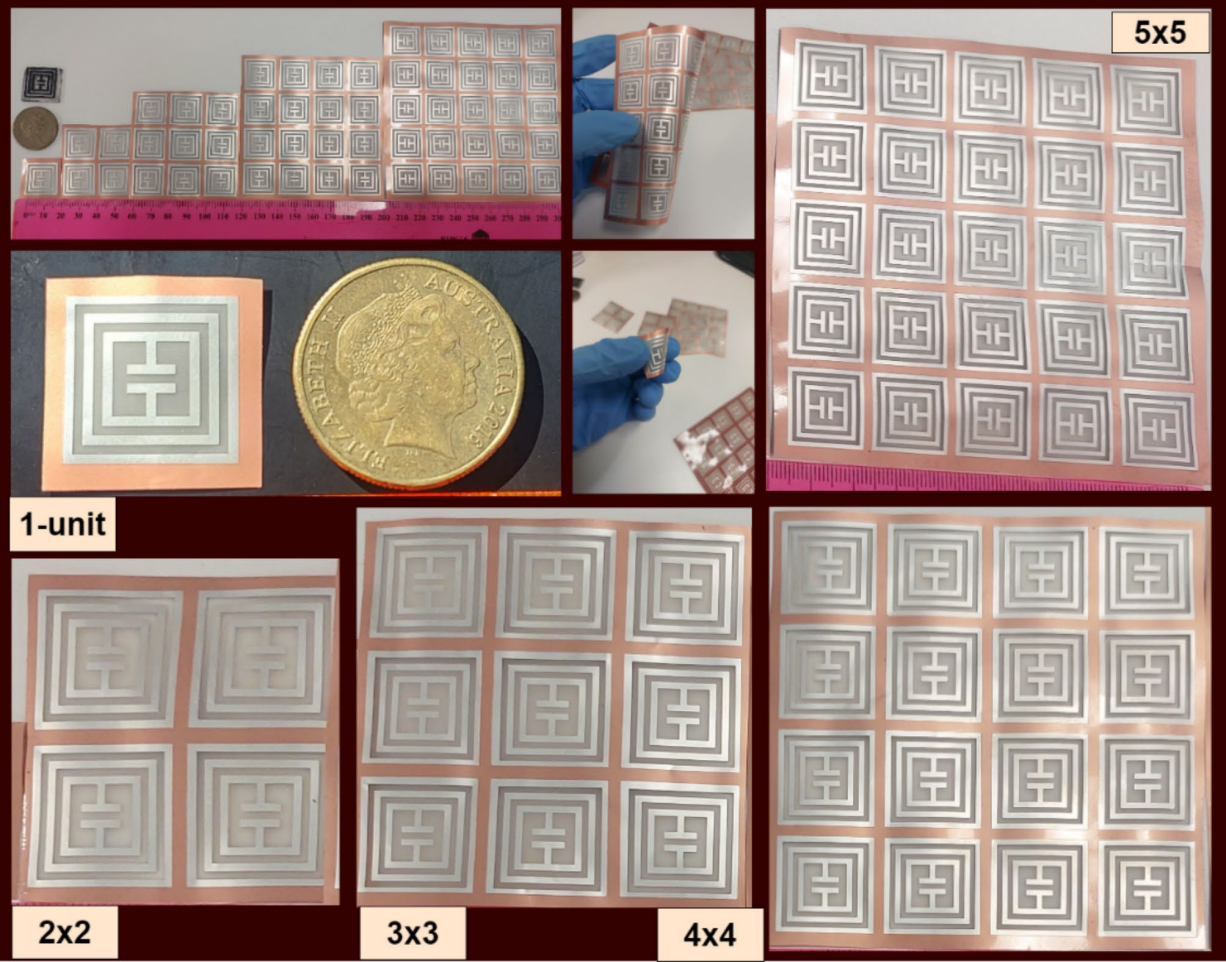
Fabricated Samples Tag-B.

The calibration process is very important to remove any extra unwanted system errors in response. After that, the calibration module is removed and SMA cables are connected to periodic antennas at port-A and port-B. In this experiment, we have considered port-A as transmitting port and port-B as receiving port. Then, the designed chipless tag is kept at far-field distance from two antennas mounted at a wooden frame to minimize interference and reflections. Then VNA is setup and readings are acquired for multiple samples of all the designed tags to see the deviation of response with any interference. The high frequency chamber foams are very crucial to absorb any unwanted and reflected signal. Figure 11 shows the array-based fabricated tag-A samples. Whereas array-based tag-B samples are illustrated in Fig. 12 respectively.

## Results

This section presents the acquired results of designed tags. Proposed work shows a novel approach towards RCS enhancement. The results acquired are interesting as a simple approach can lead us to resolve a major challenge of shorter read range in chip-free RFID tags.

### Single unit response Tag-A

**Fig. 13:**
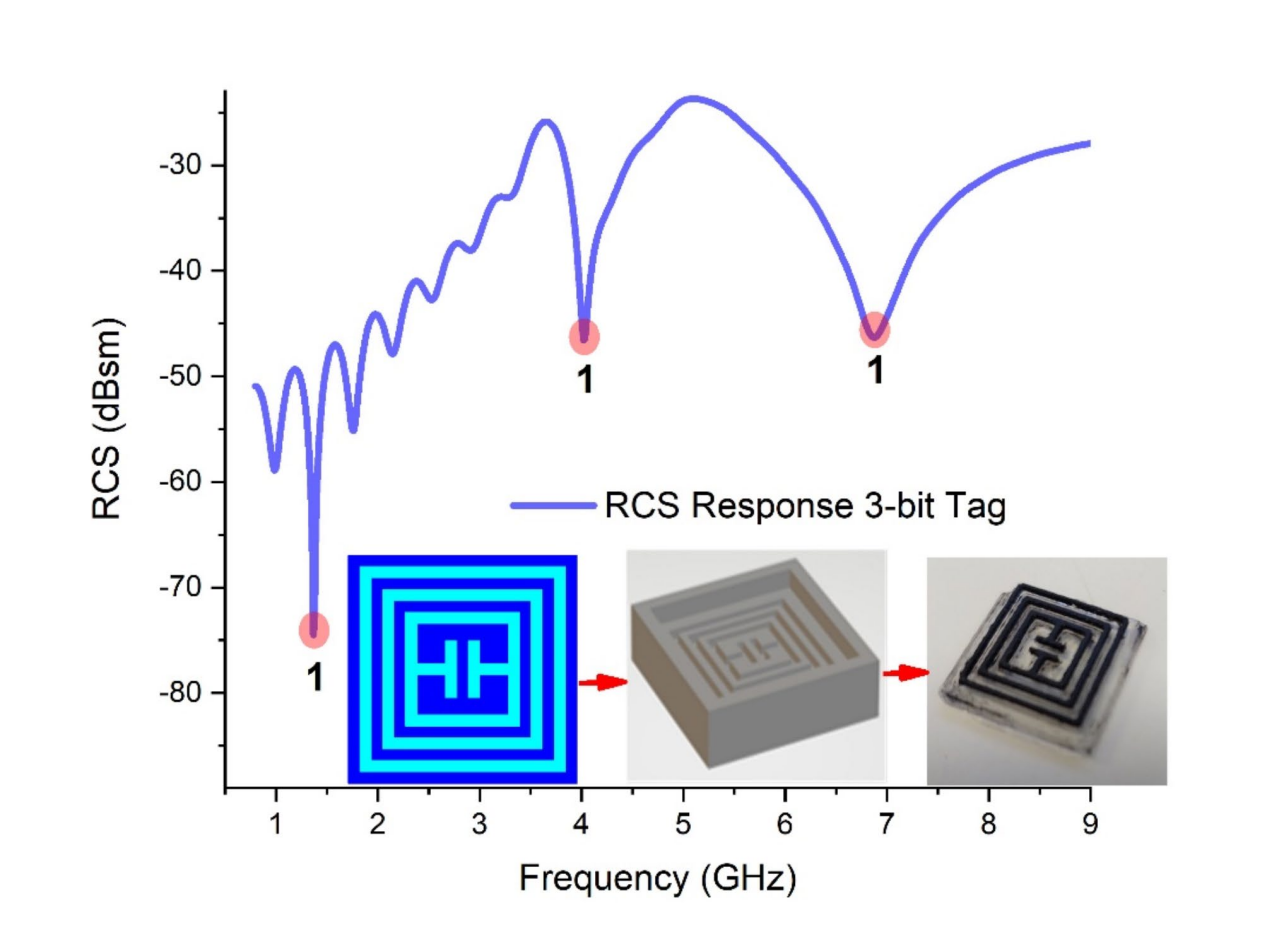
RCS Response proposed single-unit Tag-A.

The RCS response of single unit Tag-A is graphically shown in Fig. 13. The response is 3-bit resonance curve at 1.4 GHz, 4.1 GHz, and 6.9 GHz. The three dips have magnitude of RCS − 74.297dBsm, −46.575dBsm, and − 46.284dBsm respectively. The tag operates in a way that the length of the slot corresponds to resonance frequency. The longest slot resonate at smaller frequency and shortest slot corresponds to highest frequency dip.

### Array response Tag-A

**Fig. 14:**
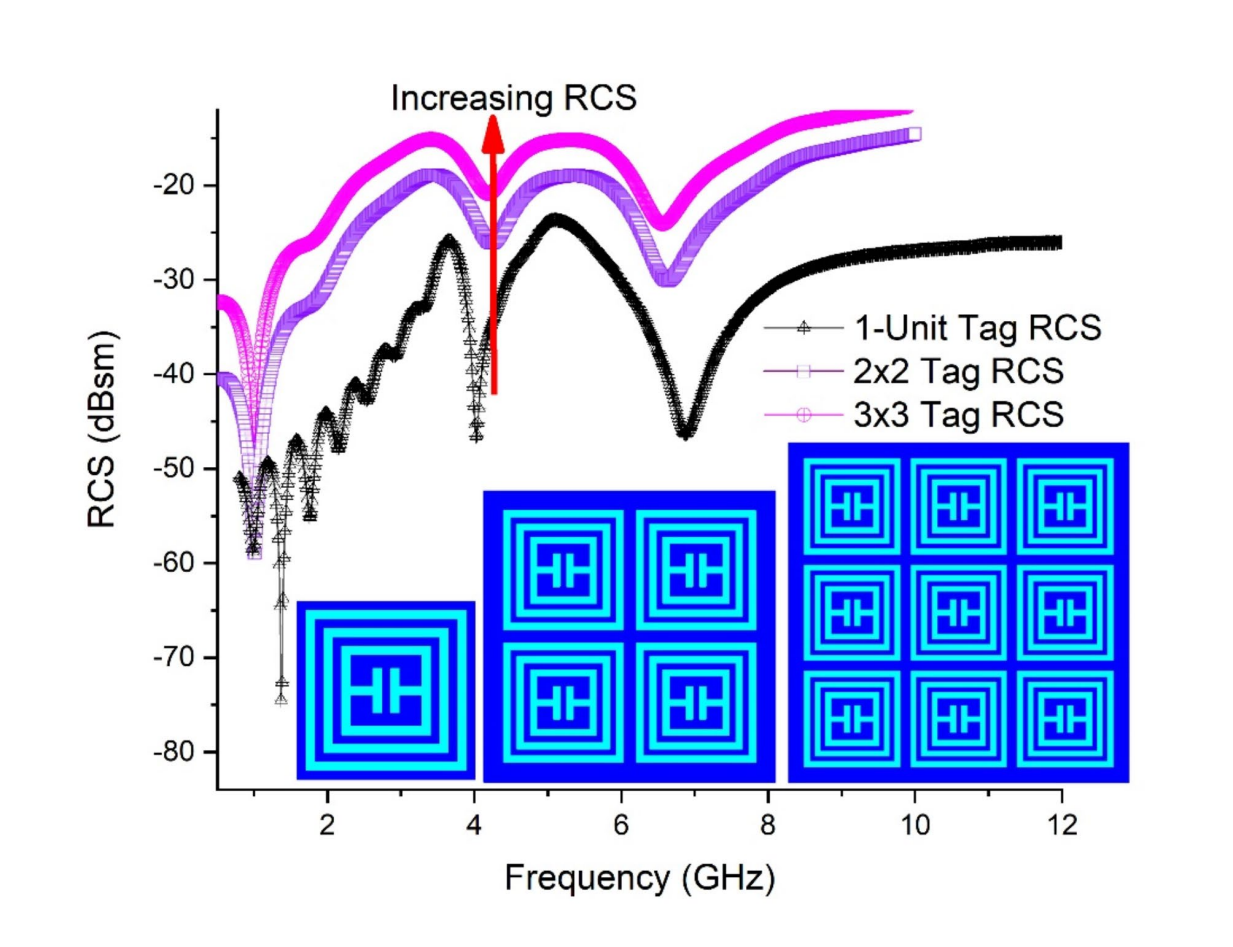
RCS Response Array Tag-A.

The array-based structures for Tag-A shows interesting response as the RCS magnitude significantly enhances with every single unit increase in array. Figure 14 shows the measured response of Tag-A for array-based tags along with 1-unit tag.

### Single unit RCS response multiples samples

**Fig. 15:**
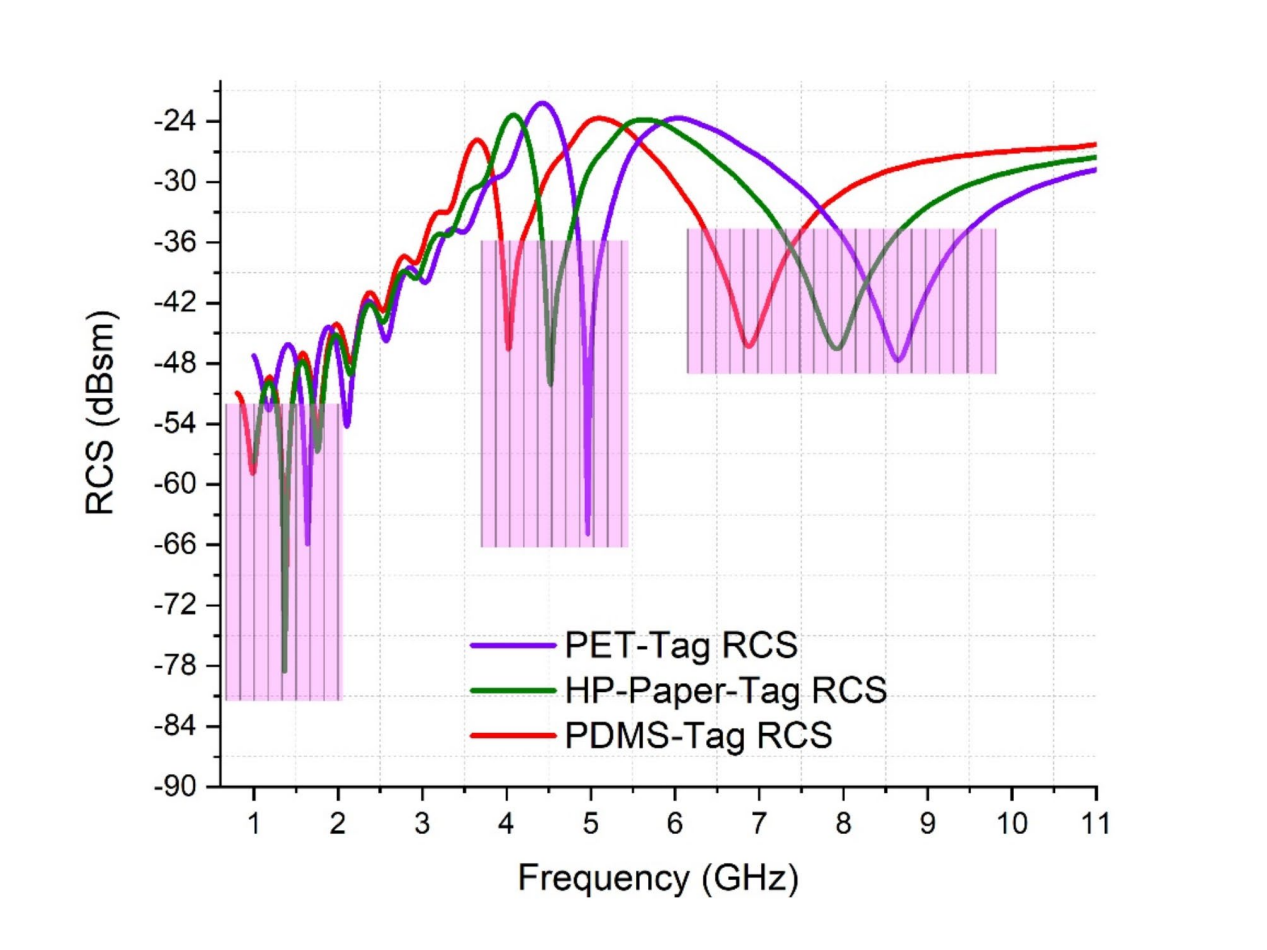
RCS Response multiple samples.

The single unit tag is further analysed and tested for PET and HP Photopaper substrate with silver nano particle-based ink. Figure 15 demonstrates graphical measured RCS response of multiple tags tested using various substrates. Tag-A is composed using PDMS having 2.88 permittivity, Tag-B is made of PET having 2.9 permittivity, whereas Tag-C is composed using HP Photopaper having 3.2 permittivity and electrical tand = 0.04. The tags responses vary based on their properties, as the permittivity varies, the response shifts towards right.

### Array response Tag-B

**Fig. 16:**
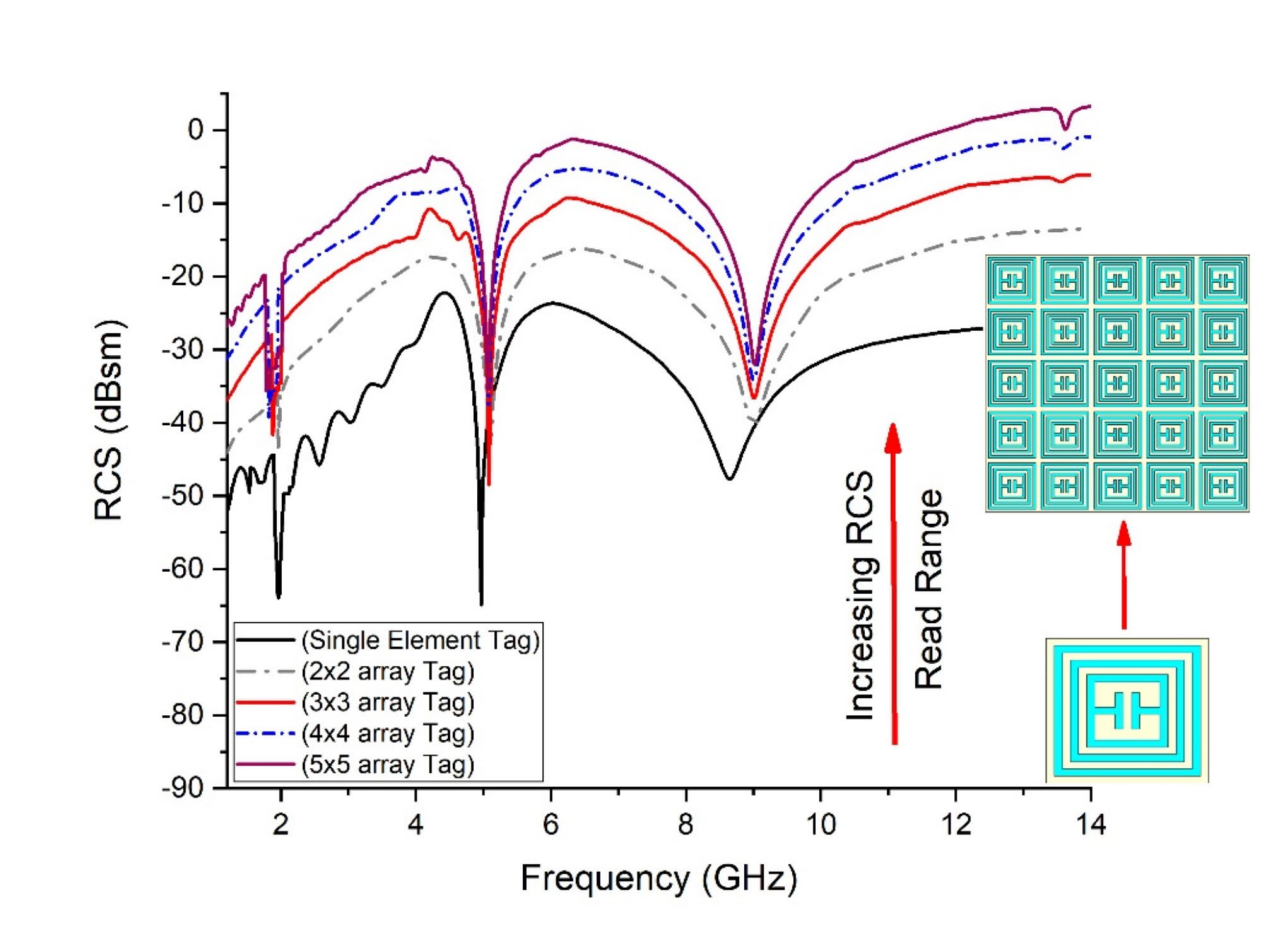
RCS Response Array Tag-B.

The tag designed using PET substrate with silver ink is referred to as ‘Tag-B’. The RCS response of designed tags is graphically illustrated in Fig. 16. The Tag-B has been designed for single element response in 20 × 20mm^2^, 2 × 2-array in 40 × 40mm^2^, 3 × 3-array in 60 × 60mm^2^, 4 × 4-array in 80 × 80mm^2^ and 5 × 5-array in 100 × 100mm^2^ dimensions. The response shows interesting and useful increment in RCS magnitude from (−59.44dBsm to −34.28dBsm).

## Discussion

This section presents the findings of proposed research. The proposed work finds useful outcomes towards RCS increment, ultimately leads us to work on low-cost read range improvement in chipless RFID tags.

### RCS Performance

**Fig. 17:**
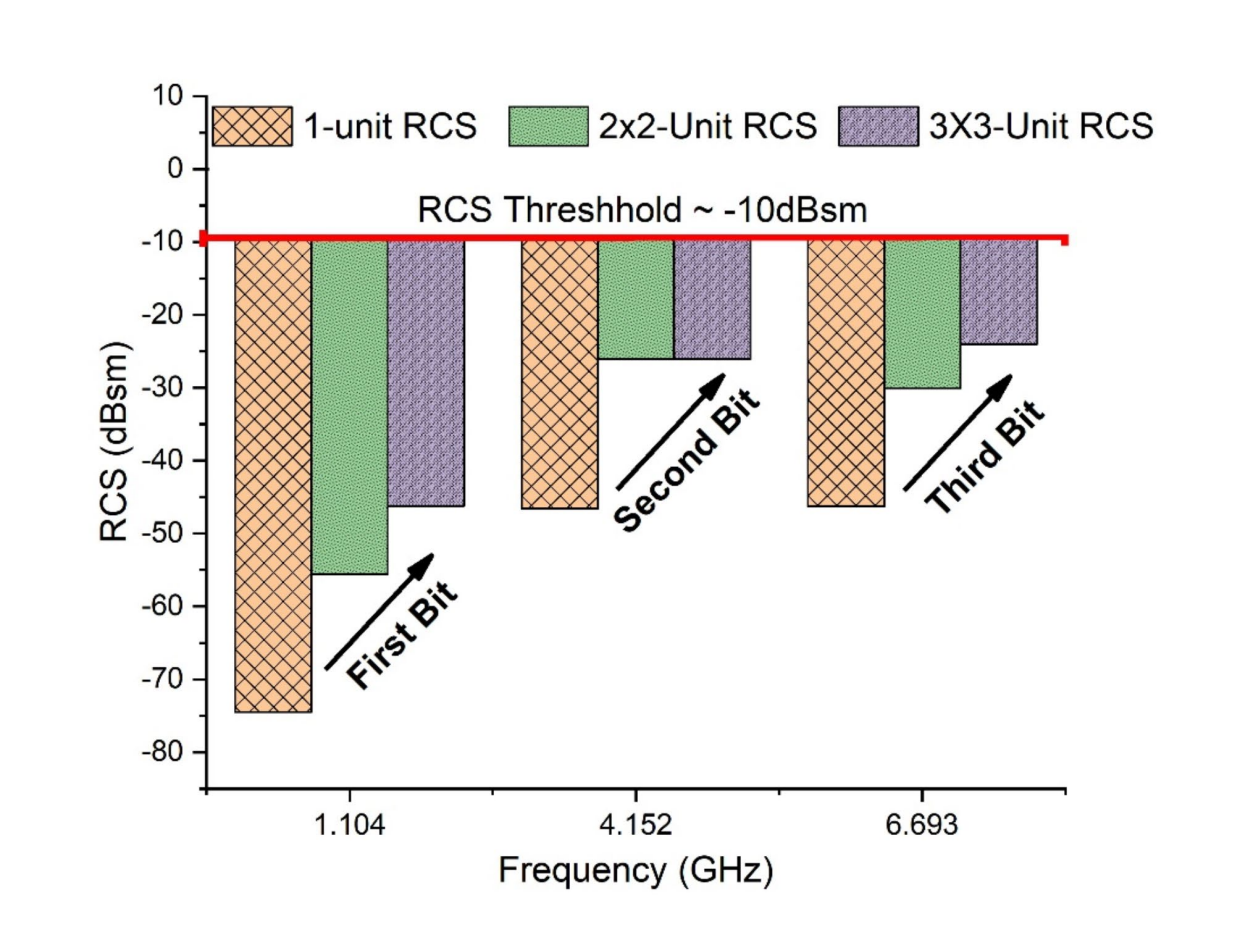
RCS performance Graph Tag-A.

The results show a reliable increment in RCS magnitudes for both Tag-A and Tag-B. Tag-A shows a shift of RCS of (−55.72dBsm to −38.35dBsm) for 2 × 2-array and (−55.72dBsm to −30.55dBsm) for 3 × 3-array, in reference from 1-unit tag. This RCS increment of 25.17dBsm shows a useful approach towards read range enhancement. Figure 17 shows RCS performance graph for Tag-A. The increment should be always reliable and below − 10dBsm threshold.

**Fig. 18:**
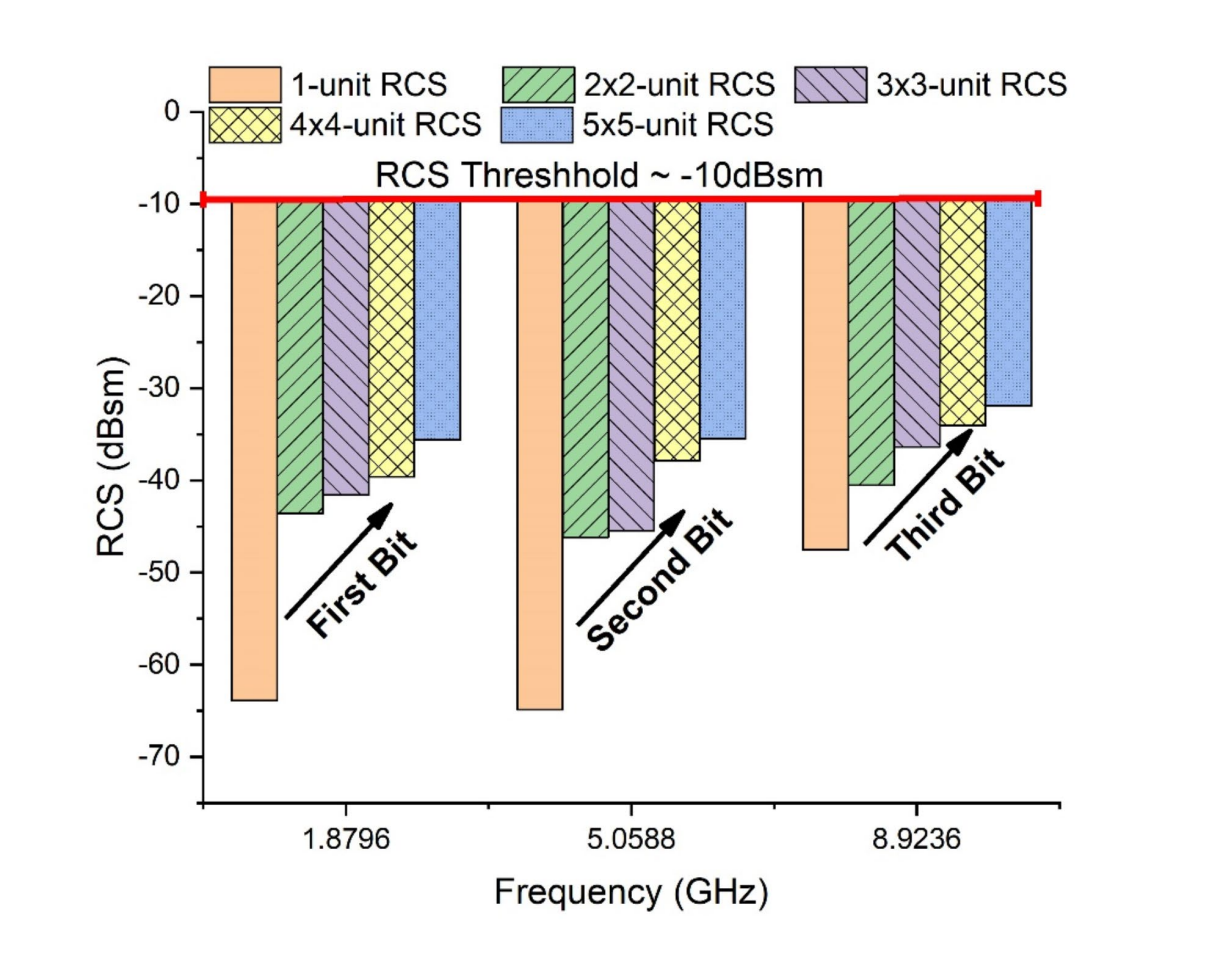
RCS performance Graph Tag-B.

Tag-B shows a shift of RCS from (−59.44dBsm to −43.09dBsm) for 2 × 2-array, (−59.44dBsm to −39.39dBsm) for 3 × 3-array, (−59.44dBsm to −36.74dBsm) for 4 × 4-array, (−59.44dBsm to −34.28dBsm) for 5 × 5-array, in reference from 1-unit tag. Figure 18 shows RCS performance graph for Tag-B with total increment of 25.16dBsm. The threshold limit is −10dBsm, below this range the tag is out of detection range.

**Fig. 19:**
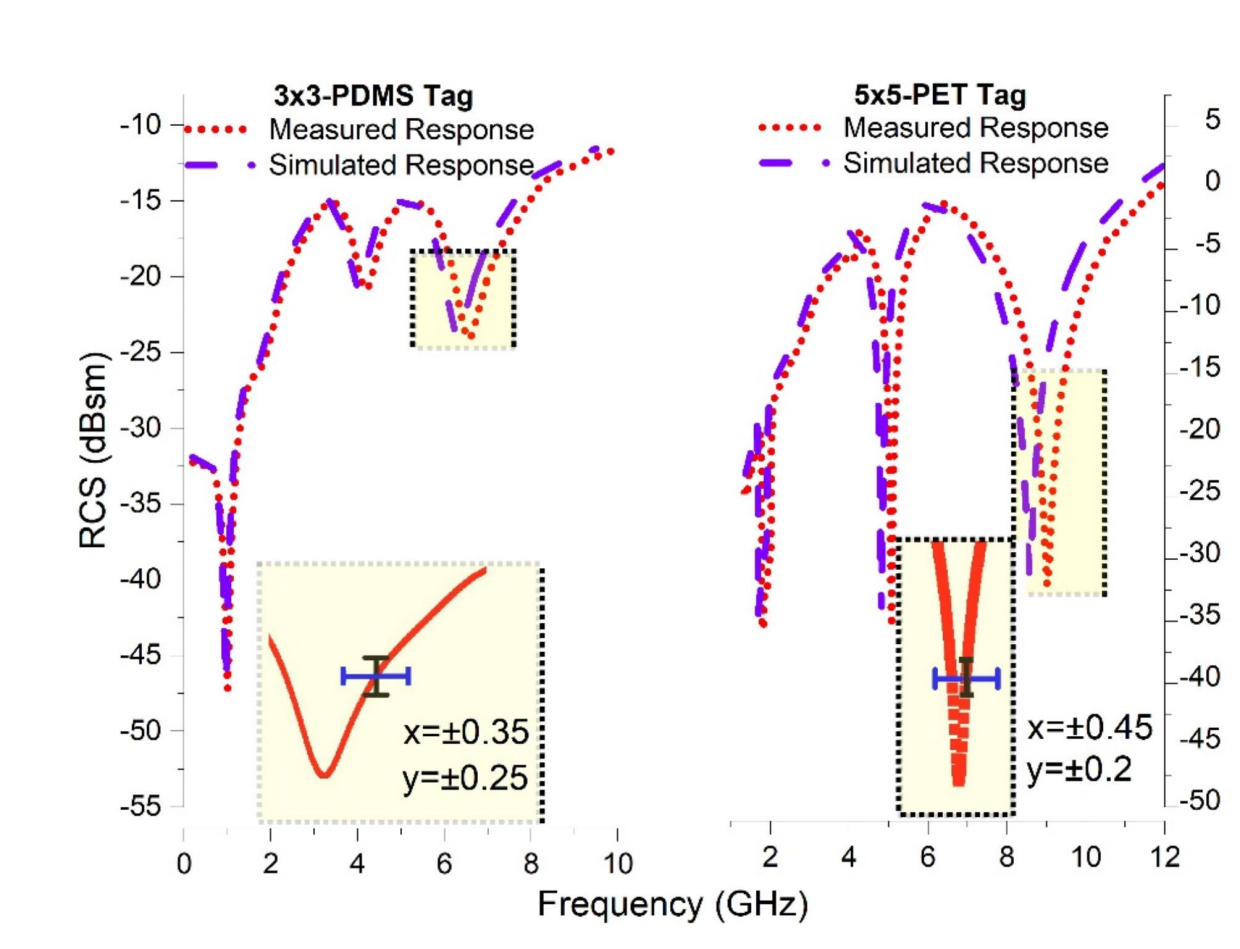
Measured and Simulated Results Analysis.

The reliability of designed tags is demonstrated by comparison of measured and simulated results. Figure 19 illustrates, a comparison for 3 × 3-array of Tag-A and 5 × 5-array of Tag-B. The reliability check of measured fabricated samples is done via testing of various samples and a probability is drawn. We have presented the possible deviation of response while testing multiple samples. For 3 × 3 PDMS tag, its (x = ± 0.35) (y = ± 0.25) whereas (x = ± 0.45) (y = ± 0.2) for 5 × 5 PET tag. Also, Table 3 provides a tabular data for RCS and resonance values for all the three bits of all proposed tags. RCS_1_, RCS_2_, RCS_3_, are RCS values for resonance dips 1,2, and 3. Resonance frequencies for three dips are referred to as f_r1_, f_r2_, f_r3_ respectively.

**Table 3:** RCS and resonance frequency, proposed tags.

Parameter	Tag-A 1-Unit	Tag-A 2 × 2	Tag-A 3 × 3	Tag-B 1-unit	Tag-B 2 × 2	Tag-B 3 × 3	Tag-B 4 × 4	Tag-B 5 × 5
RCS_1_ (dBsm)	−74.297	−58.96	−45.996	−65.889	−45.608	−41.006	−38.685	−35.497
RCS_2_ (dBsm)	−46.575	−26.05	−21.241	−64.866	−43.154	−40.545	−37.83	−35.491
RCS_3_ (dBsm)	−46.284	−30.038	−24.415	−47.575	−40.507	−36.614	−33.712	−31.838
f_r1_ (GHz)	1.3674	1.0036	0.2876	1.637	0.8054	0.028	0.039	1.0269
f_r2_ (GHz)	4.0208	4.2415	4.2143	4.965	5.096	5.082	5.082	5.069
f_r3_ (GHz)	6.8852	6.6398	6.6941	8.6743	9.016	9.002	9.0241	9.034

**Fig. 20:**
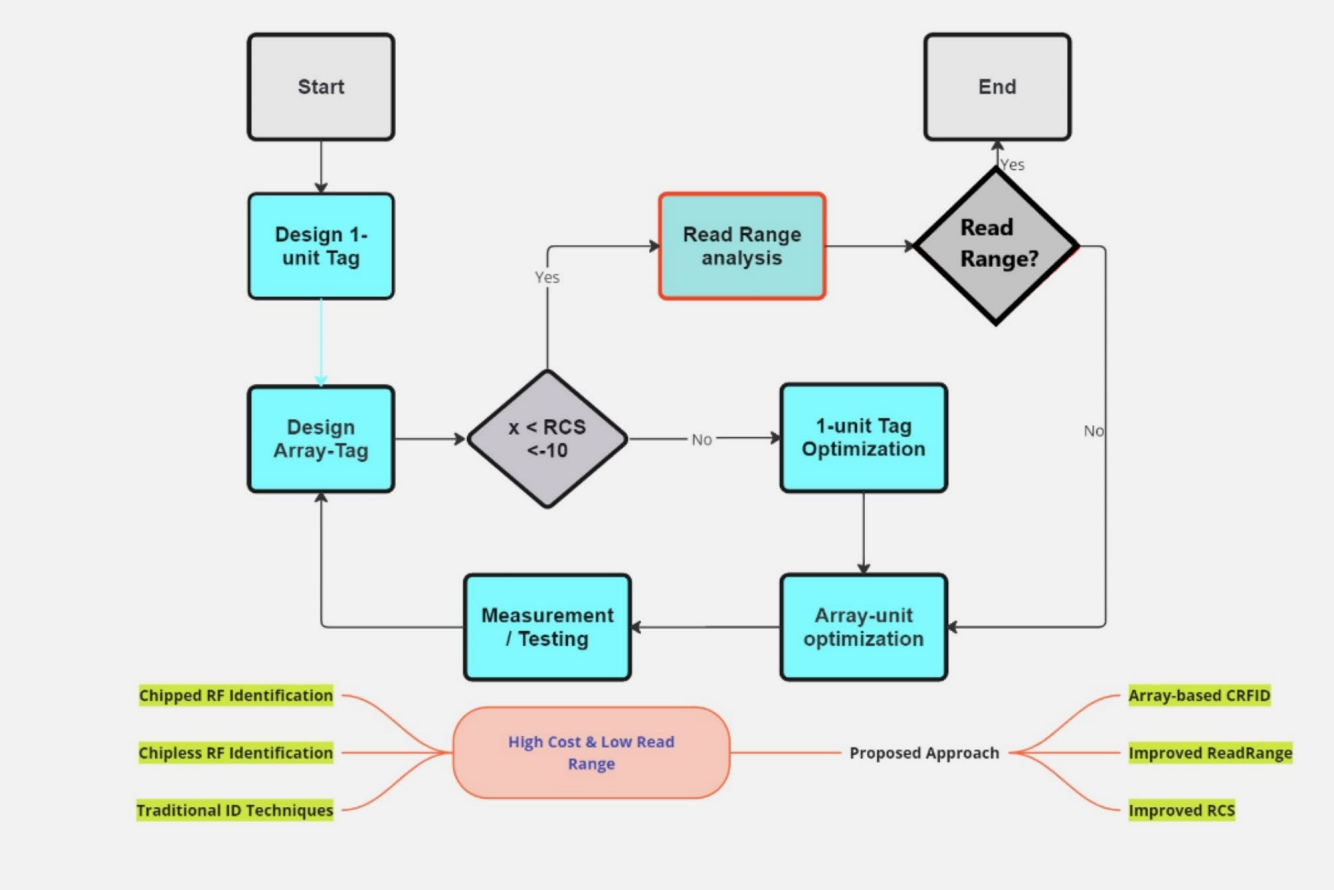
Output Oriented Approach Flow.

### Read range performance

**Fig. 21:**
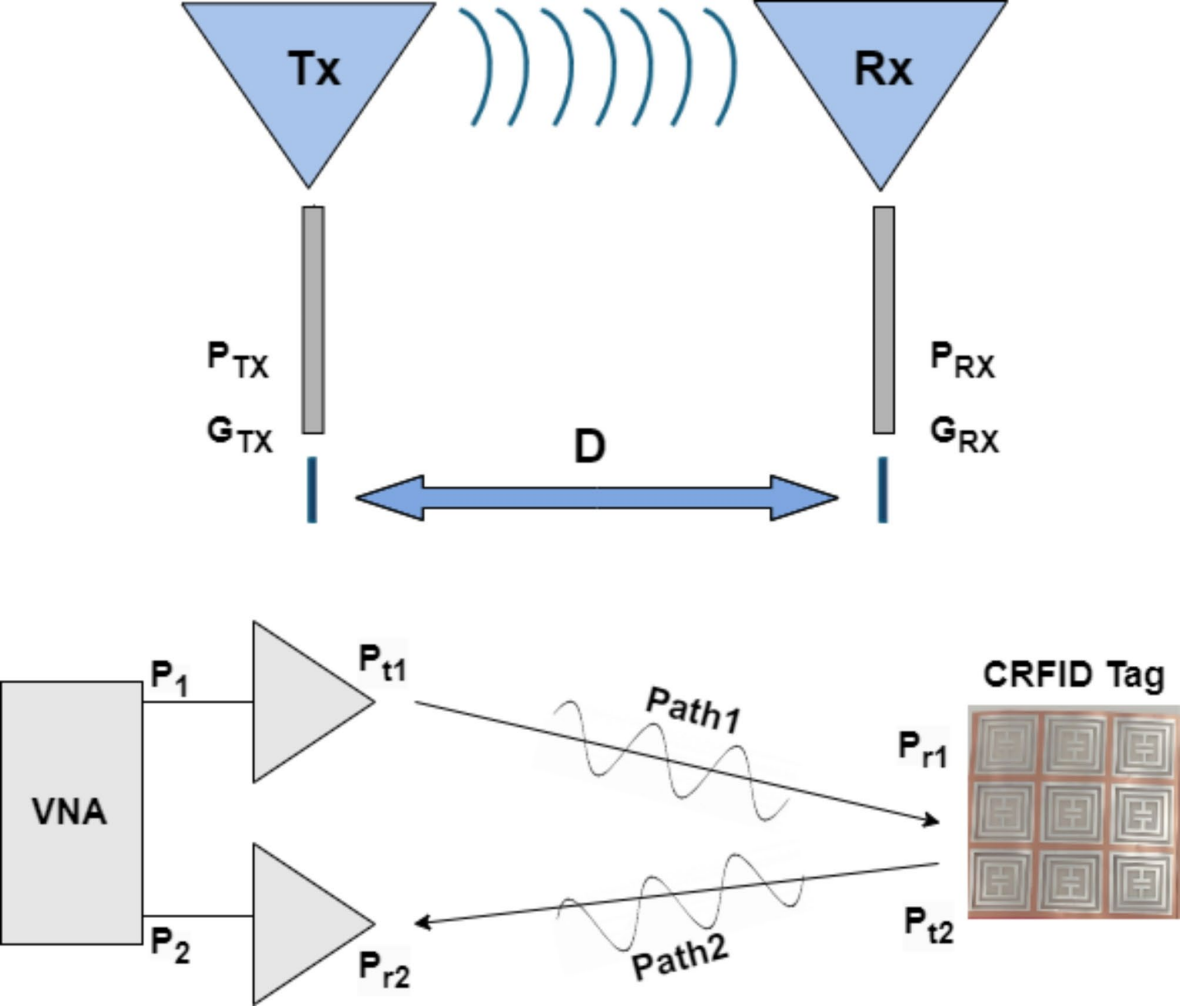
Read Range Calculation Set-up.

The very important part of this work, an outcome our examination and testing are read range performance improvement using the novel presented approach. The approach of array-based chipless tag leads us to an increment in RCS magnitude with a reliable and stable increment below threshold of −10dBsm. This leads us to an increment in read range per single unit increase of array structure from 1-unit to 5 × 5-array. Figure 20 shows the workflow of proposed approach towards read range enhancement. Figure 21 visualize the reading set-up towards read range calculations with two paths: path-I from transmitter antenna to the tag, whereas path-II is analysed from tag to the receiver antenna.

The calculations for read range using RCS responses are as follows:

- Read the tags RCS.
- Determine the gains (G_t_) and (G_r_) of transmitter and receiver antennas.
- Determine the transmitted power (P_t_).
- Calculate minimum received power (P_r_).
- Convert P_t_ and P_r_ to dBm.
- Calculate the path loss.
- Calculate the read range.

Looking at the parameters, first, we will calculate effective power reflected from tag surface towards receiver antenna P_r_. Given is P_t_=1 W, G_t_=6dBi, G_r_=6dBi. The reflected signal has 10% loss, so 90% power is reflected towards receiver. P_r_ is calculated using Eq. (1)^34^ as follows:

1 $$\:{P}_{r}=\:{P}_{T}\:\times\:\:\left(1-Loss\right)$$

$$\:{P}_{r}=\:1 W\:\times\:\:\left(1-0.10\right)=(1 W\:\times\:0.90)=0.90 W$$

Then, path loss is calculated using P_t_=1 W, P_r_=0.90 W and received signal loss = 10% via Eq. (4)^34^ as follows:

2 $$\:{P}_{t}\left(dBm\right)=10.\:{log}_{10}\:\left({P}_{t}\:(W\right)\:\times\:\:1000)$$

$$\:{P}_{t}\left(dBm\right)=10.\:{log}_{10}\:\left(1\:\times\:\:1000\right)$$

$$\:{P}_{t}\left(dBm\right)=30\:dBm$$

3 $$\:{P}_{r}\left(dBm\right)=10.\:{log}_{10}\:\left({P}_{r}\:(W\right)\:\times\:\:1000)$$

$$\:{P}_{r}\left(dBm\right)=10.\:{log}_{10}\:\left(0.90\:\times\:\:1000\right)$$

$$\:{P}_{r}\left(dBm\right)=29.54\:dBm$$

4 $$\:Path\:Loss\:\left(dB\right)=\:{P}_{t}\:\left(dBm\right)-\:{P}_{r}\:\left(dBm\right)$$

$$\:Path\:Loss\:\left(dB\right)=\:30\:\left(dBm\right)-\:29.54\:\left(dBm\right)$$

$$\:Path\:Loss\:\left(dB\right)=\:0.46\:dB$$

Finally, read range is calculated using Friis Eq. (5)^34,35^ as follows:

5 $$\:d=\:\sqrt{\frac{{P}_{t}\:{G}_{t}\:{G}_{r}\:\sigma\:\:{\lambda\:}^{2}}{{\left(4\pi\:\:{P}_{r}\right)}^{2}}}$$

Where $$\:{\prime\:}d{\prime\:}$$ is read range of chipless RFID tags, $$\:{\prime\:}{\sigma\:}^{{\prime\:}}$$ is RCS, $$\:{\prime\:}\lambda\:{\prime\:}$$ is wavelength, $$\:{{\prime\:}P}_{t}{\prime\:}$$, $$\:{{\prime\:}G}_{t}{\prime\:}$$$$\:,{{\prime\:}G}_{r}{\prime\:}$$ are transmitted power, transmitter and receiver antennas gains respectively. The performance and read range analysis is presented in tabular form in Table 4. We have used RCS at an incident RCS_i_ and opted central frequency for read range calculations as constant. For Tag-A, the read range values show a clear increase from 0.87 mm to 3.57 mm, with 2.89 mm falling in between. The ratios illustrate the relative differences in read range capabilities among the three values. Also, for Tag-B, to compare the read range values 0.29 mm, 1.01 mm, 1.28 mm, 1.47 mm, and 1.67 mm, we can analyse their relative magnitudes and ratio differences in reference to read range of single unit tag. The ratios effectively highlight the differences in read range capabilities among these five values. It can also be visualized that the read range of PDMS/MWCNTs tag is higher compared to PET/silver-ink based tag. This opens a new idealization approach towards material impact on read range of chipless RFID tags.

**Table 4:** Performance of proposed Approach.

Parameter	Tag-A 1-Unit	Tag-A 2 × 2	Tag-A 3 × 3	Tag-B 1-unit	Tag-B 2 × 2	Tag-B 3 × 3	Tag-B 4 × 4	Tag-B 5 × 5
RCS_i_ (dBsm)	−46.575	−26.05	−21.241	−64.866	−43.154	−40.545	−37.83	−35.491
RCS (m^2^)	2.2 × 10^−5^	2.5 × 10^−3^	7.5 × 10^−3^	3.24 × 10^−7^	4.89 × 10^−5^	8.89 × 10^−5^	1.64 × 10^−4^	2.84 × 10^−4^
Freq_i_ (GHz)	3.49	3.49	3.49	4.53	4.53	4.53	4.53	4.53
Read Range (mm)	0.87	2.89	3.57	0.29	1.01	1.28	1.47	1.67
Performance (mm)	-	2.02	2.70	-	0.72	0.99	1.18	1.38
Ratio	-	3.33	4.11	-	3.48	4.41	5.07	5.76

## Conclusion

The paper presents a newly developed approach for (2.70 mm) read range enhancement in chipless RFID tags. The proposed approach is proved essential in efficient performance improvement via read range enhancement. The read range values increase progressively from 0.87 mm to 3.57 mm. The percentage increases and ratios between the values highlight the relative differences in read range capabilities, with the smallest value showing the most significant relative increase to the next value. The fabrication of novel developed PDMS/MWCNTs based chipless RFID identification is presented step by step in the paper. The designed tag is capable to generate 3-bits, while staying in 20 × 20mm^2^ dimensions on a frequency band of 0.29–6.69 GHz. This is an ideal approach for read range improvement while staying in cost manageable and battery-free operation of chipless tags. Through this array-based approach, we not only achieved higher read range, but also open new pathway towards low-cost, easy to detect RFID tags for IoT and sensing networks with real-time high read range detection. The read range performance of the tags is reliable and stable, as confirmed by measurements from multiple samples, making them well-suited for use in smart sensing and IoT identification networks. In future the work can be extended towards deployment of proposed approach in real time high scale chipless Identification systems in Australia.

## Data Availability

The datasets used and/or analysed during the current study available from the corresponding author on reasonable request.
